# How do parasitic worms prevent diabetes? An exploration of their influence on macrophage and β-cell crosstalk

**DOI:** 10.3389/fendo.2023.1205219

**Published:** 2023-07-26

**Authors:** Inah Camaya, Bronwyn O’Brien, Sheila Donnelly

**Affiliations:** School of Life Sciences, Faculty of Science, University of Technology Sydney, Ultimo, NSW, Australia

**Keywords:** helminth, diabetes, macrophage, β-cells, *Fasciola hepatica*, FhHDM-1

## Abstract

Diabetes is the fastest growing chronic disease globally, with prevalence increasing at a faster rate than heart disease and cancer. While the disease presents clinically as chronic hyperglycaemia, two distinct subtypes have been recognised. Type 1 diabetes (T1D) is characterised as an autoimmune disease in which the insulin-producing pancreatic β-cells are destroyed, and type 2 diabetes (T2D) arises due to metabolic insufficiency, in which inadequate amounts of insulin are produced, and/or the actions of insulin are diminished. It is now apparent that pro-inflammatory responses cause a loss of functional β-cell mass, and this is the common underlying mechanism of both T1D and T2D. Macrophages are the central immune cells in the pathogenesis of both diseases and play a major role in the initiation and perpetuation of the proinflammatory responses that compromise β-cell function. Furthermore, it is the crosstalk between macrophages and β-cells that orchestrates the inflammatory response and ensuing β-cell dysfunction/destruction. Conversely, this crosstalk can induce immune tolerance and preservation of β-cell mass and function. Thus, specifically targeting the intercellular communication between macrophages and β-cells offers a unique strategy to prevent/halt the islet inflammatory events underpinning T1D and T2D. Due to their potent ability to regulate mammalian immune responses, parasitic worms (helminths), and their excretory/secretory products, have been examined for their potential as therapeutic agents for both T1D and T2D. This research has yielded positive results in disease prevention, both clinically and in animal models. However, the focus of research has been on the modulation of immune cells and their effectors. This approach has ignored the direct effects of helminths and their products on β-cells, and the modulation of signal exchange between macrophages and β-cells. This review explores how the alterations to macrophages induced by helminths, and their products, influence the crosstalk with β-cells to promote their function and survival. In addition, the evidence that parasite-derived products interact directly with endocrine cells to influence their communication with macrophages to prevent β-cell death and enhance function is discussed. This new paradigm of two-way metabolic conversations between endocrine cells and macrophages opens new avenues for the treatment of immune-mediated metabolic disease.

## 1 Introduction

While the term ‘diabetes’ is defined as an individual’s inability to regulate blood glucose concentrations with resultant chronic hyperglycemia, traditionally two major clinically distinct subtypes have been characterized. Type 1 diabetes (T1D) results from the complete autoimmune mediated destruction of the insulin producing beta (β) cells within the pancreatic islets (1). In contrast, type 2 diabetes (T2D) arises because the β-cell population cannot satisfy insulin demand and/or peripheral tissues are resistant to the actions of insulin (2). Although T1D and T2D have been clinically classified as separate disease entities with distinct pathogeneses, there is now increasing evidence that they share disease sequalae. Loss of β-cell function and mass is the common underlying mechanism driving the progression of both conditions, and β-cell death/dysfunction is caused by pro-inflammatory responses largely initiated and perpetuated by macrophages (3).

The incidence of T1D and T2D have been exponentially increasing in recent decades, with the global prevalence predicted to reach almost 600 million cases by 2035 (4). Such rapid increases in disease prevalence cannot be attributable to genetic modifications, and instead suggest the removal of a protective environmental factor or introduction of a predisposing agent (5, 6). Initially, epidemiological studies established a robust inverse relationship between the incidence of multiple autoimmune/inflammatory diseases, and the prevalence of endemic helminth infections (7). Subsequently, compelling results from several human and animal studies have corroborated a protective effect of helminthic infection (or their excretory/secretory [ES] molecules) against the development of both TID and T2D (8). It has been broadly proposed that this positive impact on disease outcome is mediated by the potent ability of helminths to regulate pro-inflammatory host immune responses.

Interestingly, macrophages have been identified as the key players in both the modulation of host responses during helminth infections (9), and the initiation and perpetuation of pro-inflammatory responses during diabetes development. Over the years, the central role of macrophages in β-cell differentiation and homeostasis has been well demonstrated (10). However, more recently macrophages have emerged as central players in the initiation of autoimmune insulitis (immune cell infiltration of the islets) in T1D, and as the dominant immune cell population causing intra-islet inflammation in T2D (11, 12). Macrophages are highly dynamic and adopt distinct phenotypes and functions in response to cues received from adjacent cells and the surrounding microenvironment (13, 14). Further, macrophages are among the first immune cells to traffic to the islets during the development of T1D and T2D (11, 12). Thus, it is not surprising that macrophages can, and do, communicate intimately with β-cells, and *vice versa*. This intercell crosstalk determines if the islet micro-environment becomes pro- or anti-inflammatory, thereby promoting or mitigating the development of diabetes, respectively (15, 16).

This review discusses the evidence suggesting that modulation of the host immune responses by helminths alters the interplay between macrophages and β-cells to prevent β-cell dysfunction and death, and therefore prevent the development of both T1D and T2D. Helminth infection has also been shown to alter host metabolism (17), suggesting an additional effect on cells with endocrine function, such as β-cells. Therefore, this review also explores the biological changes mediated by helminths, and their ES products, to determine if they may directly alter the communication between β-cells and macrophages, to enhance β-cell survival and function, thereby preventing the development of T1D and T2D.

## 2 β-cell dysfunction and death underpin T1D and T2D

**Figure 1:**
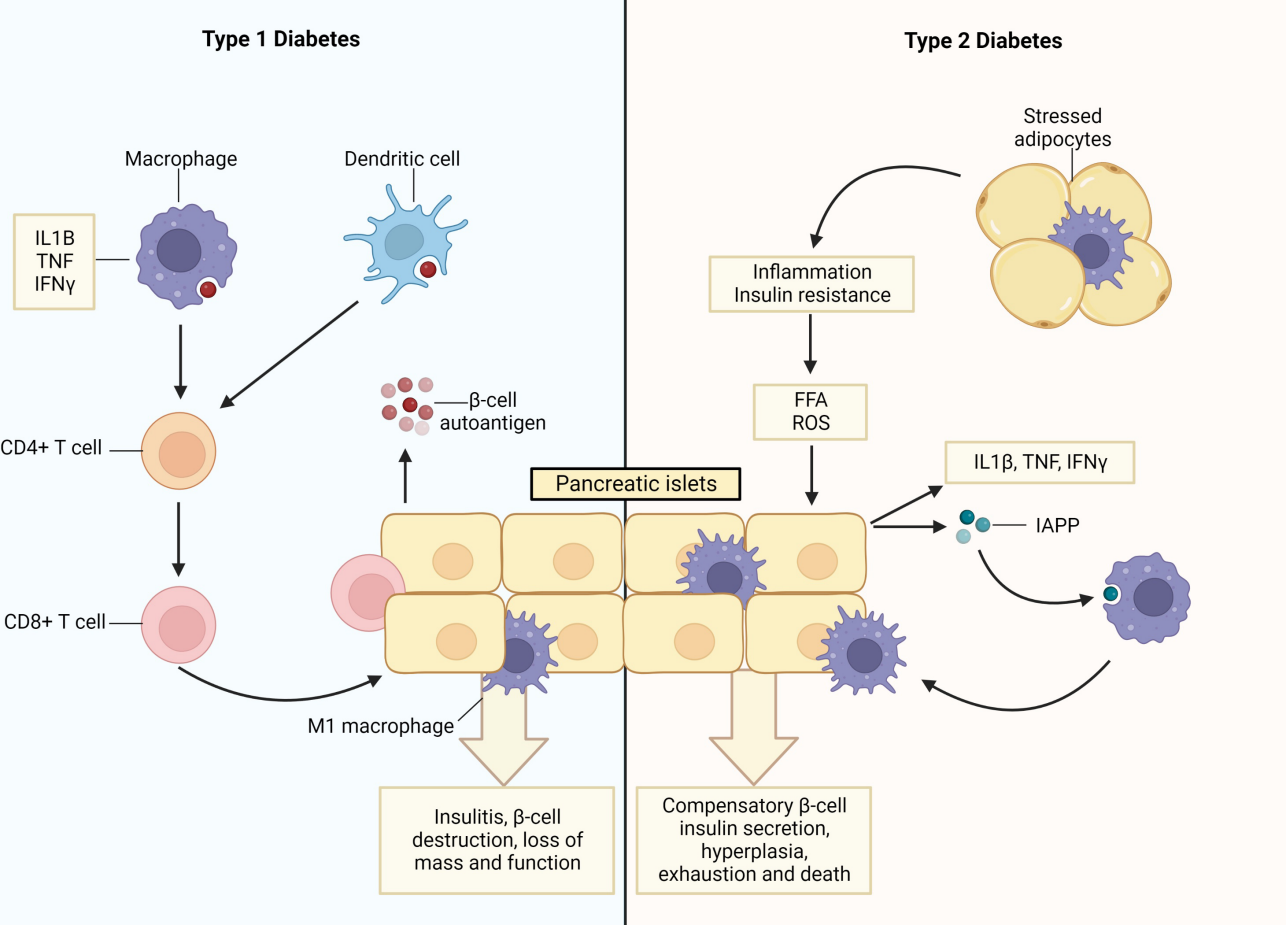
Pancreatic islet inflammation and β-cell death underpin type 1 and type 2 diabetes. In Type 1 diabetes (T1D) apoptotic β-cells are a source of auto- and neo-antigens resulting in the activation of antigen presenting cells (APCs; macrophages and dendritic cells) after their phagocytosis. This initiates inflammation and the priming of autoreactive Th1 and Th17 CD4^+^ cells, which, in turn, prime autoreactive cytotoxic CD8^+^ T cells that infiltrate the islets (termed insulitis) and destroy the β-cells. During insulitis, infiltrating macrophages and T cells secrete pro-inflammatory cytokines (IL1β, TNF and IFNγ), which increase rates of β-cell apoptosis. Additionally, stressed β-cells upregulate expression levels of MHC I/II and co-stimulatory molecules enabling them to function as APCs and also induce the activation and clonal expansion of autoreactive T-cell populations. In type 2 diabetes (T2D), there are increased levels of free fatty acids (FFAs) and inflammation. This induces β-cell endoplasmic reticulum (ER) stress and secretion of pro-inflammatory cytokines, akin to the intra-islet events observed in T1D, as well as islet amyloid polypeptide (IAPP). In turn, macrophages are recruited into the islet and islet resident macrophages become activated, both of which exhibit pro-inflammatory phenotypes. This process is exacerbated by stressed adipocytes that trigger inflammation in peripheral organs and initiate insulin resistance. Consequently, β-cells undergo compensatory hyperplasia and increased insulin secretion, which ultimately leads to β-cell exhaustion, death and T2D. Created with BioRender.com.

In T1D, β-cells are lost due to a sustained process of autoimmune destruction driven by pro-inflammatory immune cells. This destructive process progresses over several years such that at diagnosis approximately only 10-20% of β-cell mass remains (1). Within the autoimmune islet environment, dying β-cells are phagocytosed by antigen presenting cells (macrophages and dendritic cells), and autoantigens are processed and presented to autoreactive Th1 and Th17 CD4^+^ cells. Subsequently, autoreactive cytotoxic CD8^+^ T cells undergo activation and clonal expansion, and traffic to the pancreas where they infiltrate the islets and destroy β-cells (18, 19). During insulitis, infiltrating immune cells, such as macrophages and T cells, secrete pro-inflammatory cytokines (notably IL-1β, TNF, and IFNγ), which further promotes β-cell apoptosis (20, 21) (**Figure 1**). These pro-inflammatory macrophages are pivotal to T1D development as their deletion attenuates disease development (22).

While the events initiating β-cell loss are different, T2D is also ultimately an inflammatory syndrome of the islets. Indeed, conditions of overnutrition trigger inflammation and insulin resistance, along with elevated levels of inflammatory factors, such as circulating glucose and free fatty acids (FFA) (23). This induces β-cell endoplasmic reticulum (ER) stress and β-cell secretion of pro-inflammatory cytokines/chemokines (such as IL-1β, TNF, and IFNγ) as well as islet amyloid polypeptide (IAPP). In turn, this pro-inflammatory milieu leads to the recruitment of macrophages to the islet, akin to insulitis development in T1D, in addition to the activation of islet resident macrophages. These macrophage populations exhibit a pro-inflammatory M1-like phenotype and release the same inflammatory cytokines/chemokines characteristic of T1D development (24, 25). This inflammatory sequalae is exacerbated by stressed and/or necrotic adipocytes, which similarly cause the recruitment of pro-inflammatory macrophages, and reductions in anti-inflammatory macrophage and regulatory T cell populations within the adipose tissue. This, in turn, triggers inflammation in organs targeted by insulin and initiates insulin resistance. To compensate for these adverse metabolic events, β-cells produce and secrete increased amounts of insulin, resulting in β-cell hyperplasia, stress, exhaustion, and ultimately death (26–28). Collectively, these processes lead to the perpetuation and maintenance of a pro-inflammatory environment within the islet, ultimately culminating in β-cell dysfunction, death and T2D (2) (**Figure 1**).

## 3 β-cells are active participants in their own destruction

The destructive consequences of insulitis on β-cells led to the long-held paradigm that β-cells were passive victims of the detrimental pro-inflammatory islet environments characteristic of T1D and T2D. However, this notion has been challenged by more recent evidence that β-cells are active participants in their own demise (1, 29).

The neonatal phase of pancreatic remodeling is characterized by waves of β-cell proliferation, apoptosis and neogenesis. While this is intended as a physiological phenomenon, the process can lead to the generation of β-cell autoantigens, and the stimulation of autoreactive T-cells, which play a major role in the β-cell destruction that leads to diabetes progression. However, the frequencies of these autoreactive T-cell populations in the peripheral blood of T1D patients is comparable to those observed in healthy individuals. This suggests that the activation of insulitis, which underpins the initiation and progression of diabetes, requires the immune tolerance to β-cell autoantigens to be broken (1, 30).

Analyses of human insulitis lesions have suggested that signals released from stressed β-cells precedes, and putatively initiates, the development of insulitis (31). This sequence of events is recapitulated in a humanized mouse model of diabetes, in which the presence of autoreactive T-cells alone was insufficient to induce disease, which was triggered only when β-cells were stressed by the addition of the diabetogenic agent, streptozotocin (STZ) (32). Furthermore, immunosuppressive therapies that solely target T-cells fail to provide long-term protection against T1D as they do not typically address the underlying loss of β-cell immune tolerance (33). Thus, it has now been proposed that activation of the cellular stress response in β-cells due to their metabolic activities promotes cell death pathways, and participates in the initiation and amplification of inflammation and the active destruction of β-cells in T1D (34). Likewise, β-cells undergo metabolic stress in T2D due to overnutrition and inflammation that causes β-cell compensatory insulin secretion, followed by exhaustion and ultimately death (2).

Beta cells rapidly respond to the minute-to-minute fluctuations in blood glucose levels by tightly regulating insulin secretion. Such high metabolic demand to produce and secrete insulin can render the β-cells susceptible to exceeding ER protein folding capacity, in turn leading to accumulation of misfolded proteins and ER stress (35). This can dysregulate several processes, such as inhibition of β-cell function, induction of apoptosis and activation of cytosolic post-translational modification (PTM) enzymes (36), which can generate a group of neoantigens called hybrid insulin peptides (HIPs) by covalently linking insulin peptides to β-cell granule peptides, such as insulin c-peptide and IAPP. These HIPs contribute to autoimmune responses and ultimately β-cell destruction, as they are recognized by autoreactive CD4^+^ T cells in both mouse models and human patients of T1D (37, 38). Beta cells from diabetic patients also express increased levels of MHC-I and MHC-II molecules (HLA-I and HLA-II, respectively, in humans), the latter being conventionally expressed only by antigen presenting cells. This enables β-cells to present peptides (notably autoantigens) to CD8^+^ and CD4^+^ T cells, respectively (39, 40). Moreover, immune cells have direct access to islets through the dense network of islet vasculature, which can be particularly detrimental in the presence of activated pro-inflammatory immune cells and cytokines that induce β-cell stress and apoptosis (34, 41).

These β-cell characteristics make them vulnerable to destruction, and this is exacerbated in the pathogenic conditions of T1D and T2D, in which there are increased levels of pro-inflammatory cytokines, reactive oxygen species (ROS) and nitric oxide (NO), which are especially detrimental since β-cells have limited antioxidant defense capabilities (42, 43), and are highly sensitive to cytokine mediated damage. The central pro-inflammatory cytokines, IL-1β, TNF and IFNγ, have been shown to inhibit β-cell function and activate apoptotic pathways in β-cell lines, human islets, and rodent models (34, 44). In turn, this inflammatory milieu stimulates the β-cells themselves to secrete pro-inflammatory cytokines and chemokines, thereby actively contributing to their own destruction (45). Indeed, β-cells of diabetic animals and within the islets of T2D patients were observed to produce IL-1β (46). This is corroborated by *in vitro* studies, wherein cultured human islets exposed to high glucose were observed to secrete IL-1β, with production increasing due to autocrine feedback (47).

Furthermore, β-cells produce several chemokines: (i) C-C ligand 5 (CCL5), also named RANTES, (ii) C-X-C motif chemokine ligand 10 (CXCL10), also called IP-10, and (iii) C-C ligand 2 (CCL2), also termed monotype chemoattractant protein or MCP1 (34). CXCL10 and CCL5 are secreted by murine islets, β-cell lines, and cultured human islets after exposure to IL-1β, TNF, and IFNγ. Both chemokines attract activated immune cells to the islets (48, 49), and CXCL10 has been shown to exert direct toxicity on the β-cells (50). Similarly, CCL2 is produced by human and murine islets in response to IL-1β and can be induced *in vivo* by environmental triggers (such as viral infections), causing inflammation and macrophage recruitment (51–53).

**Figure 2:**
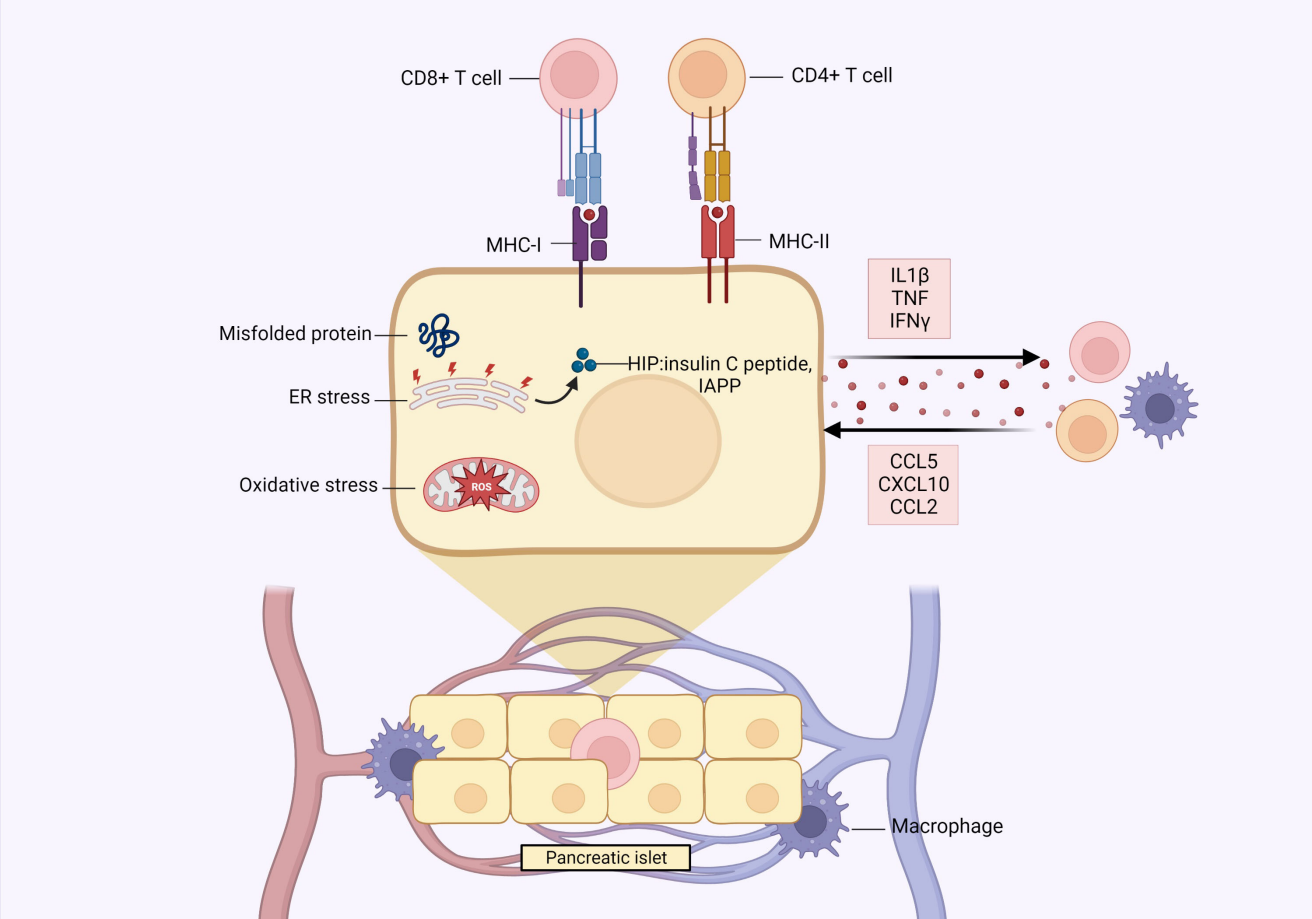
Pancreatic β-cells play an active role in their own destruction. The high metabolic demand of insulin production/secretion can render β-cells vulnerable to exceeding endoplasmic reticulum (ER) protein folding capacity, which can lead to accumulation of misfolded proteins and ER stress. This results in dysregulation of β-cell function, induction of apoptosis and generation of hybrid insulin peptides (HIPs), such as insulin c-peptide and islet amyloid polypeptide (IAPP), which can be recognized by autoreactive immune cells, thereby contributing to β-cell destruction. Beta cells also express MHC-I and MHC-II, the latter conventionally only being expressed by bonafide antigen presenting cells to present peptides to CD8^+^ and CD4^+^ T cells, respectively. The dense network of islet vasculature also allows immune cells direct access to the islets, which can be especially detrimental under diabetic conditions wherein immune cells generally exhibit pro-inflammatory activities. In a diabetic environment, there are elevated levels of reactive oxygen species (ROS) and pro-inflammatory cytokines, to which β-cells have suboptimal defense mechanisms due to their limited production of antioxidant enzymes and sensitivity to cytokine damage. In response to this inflammation, β-cells themselves secrete pro-inflammatory cytokines (IL1β, TNF, IFNγ) and chemokines (CCL5, CXCL10, CCL2), which contribute to β-cell destruction through self-toxicity or activation/recruitment of immune cells, such as macrophages. Created with BioRender.com.

Collectively, these β-cell vulnerabilities, along with the signals they release under conditions of stress within the islet microenvironment, can create a self-perpetuating cycle of β-cell destruction, which is exacerbated in conjunction with pro-inflammatory immune cells (**Figure 2**).

## 4 Crosstalk between macrophages and β-cells predetermines the fate of β-cells

Macrophages constitute a heterogeneous and dynamic immune cell population whose phenotype and function are highly dependent on the tissue microenvironment. Macrophages can derive from embryonic hematopoietic precursors and inhabit specific tissues, in which they actively participate in maintaining homeostasis under physiological conditions. Following pathogen infection, tissue damage, release of inflammatory mediators, or metabolic cues, circulating monocytes are recruited in different tissues where they differentiate into resident macrophages. Here, stimuli in the surrounding micro-environment induce macrophages to adopt pro-inflammatory or anti-inflammatory phenotypes and functions, thereby mediating inflammatory/autoimmune or homeostatic/reparative responses, respectively (22, 54).

**Figure 3:**
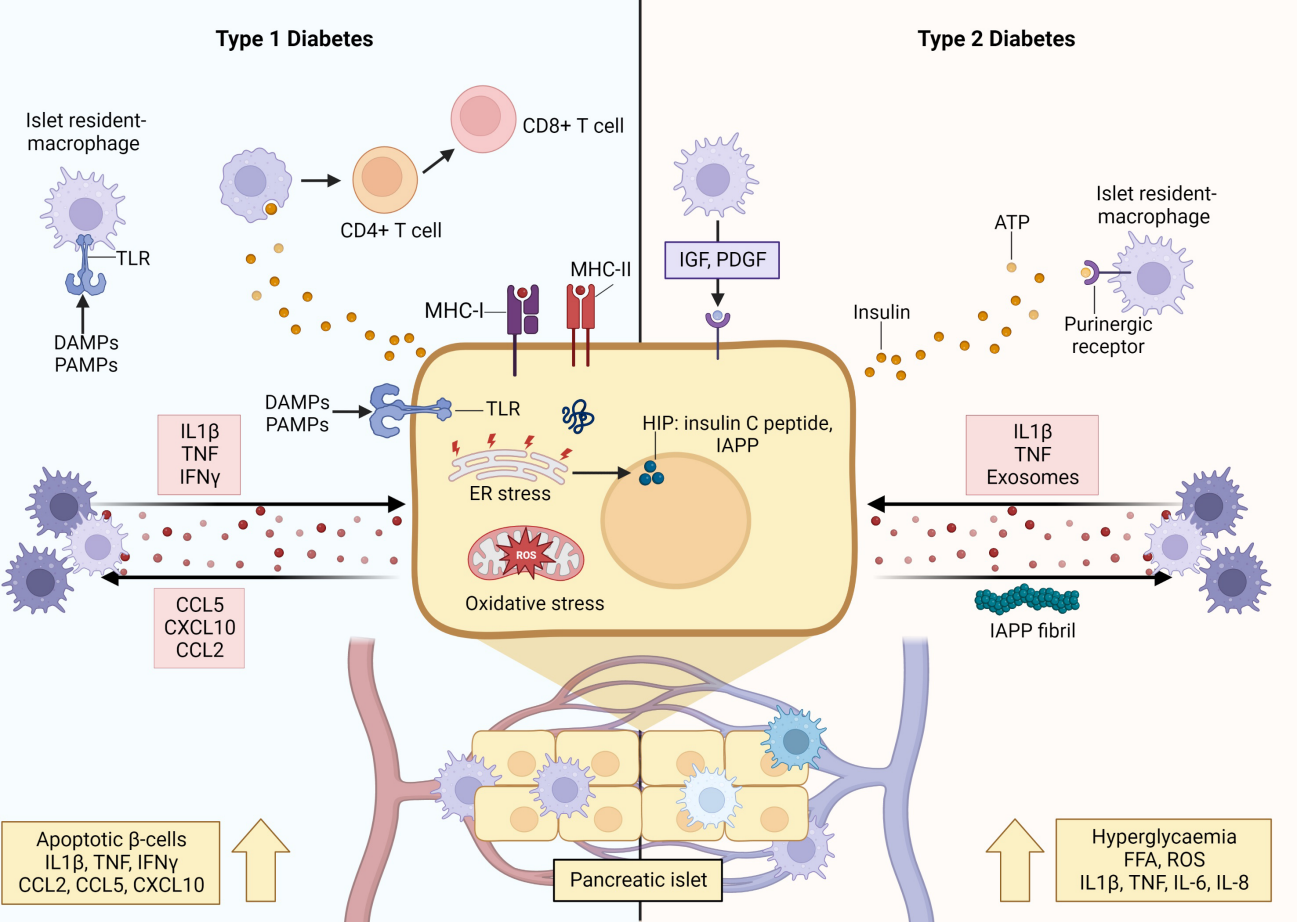
Crosstalk between macrophages and β-cells orchestrates inflammation and β-cell destruction in type 1 and type 2 diabetes. When physiological homeostasis is disrupted by inflammatory signals, communication between β-cells and macrophages drive β-cell dysfunction and apoptosis. In the initiating stages of T1D, resident islet macrophages exhibit a pro-inflammatory M1-like phenotype characterized by secretion of pro-inflammatory cytokines, upregulated MHC-II expression, and increased antigen presentation capacity. The β-cells also secrete pro-inflammatory cytokines and chemokines that recruit monocyte-derived macrophages with pro-inflammatory phenotype into the islet. Furthermore, β-cells also secrete insulin vesicles containing immunogenic peptides that are taken up by macrophages for presentation to autoreactive T cells, and express MHC-II, allowing them to present autoantigens as well. Together, these interactions reinforce and amplify β-cell apoptosis and destruction underlying the irreversible loss of β-cell mass leading to T1D. During T2D, hyperglycaemia and overnutrition cause β-cell hyperactivation and hyperplasia leading to functional changes and increased metabolic rates (ER and oxidative stress) within the β-cells. These changes are sensed by resident islet macrophages via uptake of insulin-containing vesicles, or through overactivation of purinergic receptors by ATP. Furthermore, β-cells secrete islet amyloid polypeptide (IAPP), which can aggregate to form plaques and fibrils that skew macrophages to a destructive pro-inflammatory M1-like phenotype. Islet macrophages do not purely exhibit M1 features and can display anti-inflammatory M2 activity, wherein they exhibit increased expression levels of reparative factors, such as IGF-1, that promote β-cell survival and proliferation. Created with BioRender.com .

Macrophages are among the first cells to traffic to the pancreas under both physiological and diabetogenic conditions. This is because macrophages play central roles during neonatal islet remodeling, which is characterized by waves of β-cell differentiation, proliferation, and apoptosis, due to their potent phagocytic abilities. After taking up residence in the islets, macrophages remain in intimate contact with the β-cells, where they act as exquisite sensors of the function and viability of β-cells (15). Through a two-way exchange of signals, such as cytokines and chemokines, hormones and growth factors, insulin-containing vesicles and exosomes, and metabolites, macrophages are highly responsive to β-cell activities, and the composition of their secretions is modulated accordingly to promote β-cell survival and metabolic activity (under physiological cues). On the other hand, macrophage/β-cell crosstalk can drive β-cell dysfunction and apoptosis when physiological equilibrium is disturbed by the inflammatory signals present during the development of T1D and T2D (16) (**Figure 3**).

In the context of T1D, the populations of resident islet macrophages in both animal models and humans display an pro-inflammatory phenotype, characterized by the secretion of cytokines (such as IL-1β and TNF), upregulated expression of MHC-II and increased antigen presentation capacity, and decreased phagocytosis of apoptotic β-cells (55–57). Decreased uptake of dying β-cells by macrophages and/or increased rates of β-cell apoptosis leads to an accumulation of dying β-cells, and their progression to necrosis, during the intended physiological process of neonatal islet remodeling. Also, after the phagocytosis of apoptotic β-cells, macrophages secrete increased levels of pro-inflammatory IL-12 and decreased levels of IL-10, which contradicts the expected anti-inflammatory pro-resolving response (58–60). Aside from this cytokine profile, intra-islet macrophages secrete NO and chemokines, and express chemokine receptors (notably CCR5, CXCR3 and CCR8) which further recruits and activates other pro-inflammatory immune cells (61, 62). These phenomena modulate the crosstalk between β-cells and macrophages to trigger the initiation of disease.

Furthermore, monocyte-derived macrophages are among the first, and most abundant, immune cells to traffic into the islet in both murine models and human patients (11, 63), and it is hypothesized that this initial influx is induced by increased levels of CCL2. Indeed, CCL2 overexpression in murine β-cells promoted monocyte recruitment to islets, infiltration, and β-cell destruction (53). Moreover, macrophages express C-C chemokine receptor 2 (CCR2) to which CCL2 can bind, thereby inducing macrophage secretion of the pro-inflammatory mediators, IL-1β, TNF, IL-12, and CXCL10. These islet infiltrating macrophages are predominantly characterized as a pro-inflammatory phenotype and release potent inflammatory mediators (such as IL-1β and CCL2) that promote β-cell apoptosis (64). Simultaneously, signals from apoptotic β-cells (IL-1β, CCL5, CXCL10, CCL2) amplify/maintain this pro-inflammatory phenotype (65).

This inflammatory environment can be exacerbated by priming factors that mediate crosstalk between macrophages and β-cells. For instance, β-cells and islet resident macrophages express toll-like receptors (TLRs), which are activated by pathogen activated molecular patterns (PAMPs) and damage associated molecular patterns (DAMPs) released due to viral infection and other tissue damaging agents. This stimulates the secretion of pro-inflammatory cytokines (such as IL-1β and TNF) that can reinforce and amplify a pro-inflammatory function/phenotype of islet resident macrophages and activate apoptotic pathways within β-cells (66). As aforementioned, stressed and/or apoptotic β-cells release pro-inflammatory signals, which are endocytosed by macrophages, thereby perpetuating the cycle of inflammation. Furthermore, insulin vesicles secreted by β-cells contain immunogenic peptides, which can also be taken up by resident M1-like macrophages for processing and presentation to autoreactive T cells. Aside from the uptake of secreted vesicles, intracellular cargo from apoptotic β-cells can be transferred to macrophages during phagocytosis (64). Additionally, under the pro-inflammatory conditions β-cells begin to express MHC-II, which enables them to present autoantigens (notably insulin neoantigens) to antigen presenting cells (40). Ultimately, the activities of β-cells and macrophages culminate in the activation and clonal expansion of autoreactive T cells, which drive the accelerated and irreversible loss of β-cell mass leading to T1D.

Similarly, under T2D conditions the crosstalk between macrophages and β-cells plays a major initiating role in establishing β-cell dysfunction, and a pro-inflammatory macrophage phenotype. In T2D, overnutrition and transient hyperglycemia induce β-cell hyperactivation and hyperplasia to satisfy the increased insulin demand (23). Aside from increased insulin secretory rates, β-cells undergo multiple functional changes induced by the nutrient rich environment and increased metabolic rates, including the production of pro-inflammatory mediators, such as NO and ROS (67). As islet macrophages constantly probe their microenvironment for signals, they sense these fluctuations in β-cell activity, via uptake of insulin-containing vesicles or through overactivation of their purinergic receptors by ATP. This can lead to desensitization and downregulation of homeostatic cues, as supported by reports of decreased purinergic receptor gene expression in T2D macrophages (68). Islet resident macrophages also contribute to islet vascular remodeling that supports compensatory hyperinsulinemia (69). While this may be advantageous in the early phases of compensatory prediabetes, this advantage is nullified by the chronic background of inflammation in which β-cell hyperactivation and dysfunction are amplified, ultimately driving β-cell exhaustion and loss.

Moreover, in T2D patients, β-cells secrete IAPP along with insulin (70). The former can aggregate to form plaques and fibrils, which skew islet macrophages to a pro-inflammatory phenotype and induce IL-1β secretion in circulating monocyte-derived macrophages to facilitate their recruitment into the islet (71). Additionally, this inflammatory dialogue is amplified by increased levels of FFAs (i.e. overnutrition) and pro-inflammatory cytokines (IL-1β, TNF, IL-6, and IL-8) (72, 73). Collectively, this environment induces β-cell dysfunction and eventual death (due to the actions of inflammatory mediators and metabolic exhaustion) (15). In addition, pro-inflammatory M1-like macrophages in T2D mice were found to secrete exosomes containing the microRNA, miR-212-5p, that can be taken up by β-cells, in turn inhibiting SIRT2-mediated Akt activation and subsequently impairment of glucose stimulated insulin secretion (74).

Importantly, islet macrophages do not exclusively display an M1-like phenotype, expressing both pro-inflammatory (CD68) and anti-inflammatory (CD163) markers (75). Furthermore, it has been observed that in animal models of T2D, islet macrophages undergo local expansion and specific transcriptional changes, with two distinct subsets identified: intra-islet (CD11c^+^) and peri-islet (CD11c^-^) macrophages. The former was found to inhibit β-cell function putatively through increased uptake of β-cell secreted insulin vesicles in a cell-to-cell contact dependent manner. On the other hand, both macrophage populations promoted compensatory β-cell proliferation (76). It has also been reported that in response to increased β-cell death in induced murine models of diabetes (STZ alone or in combination with a high fat diet) and in a genetic mouse model of T2D, islet macrophages exhibited increased expression levels of insulin like growth factor 1 (IGF-1), concomitant with decreased expression of IL-6 and TNF, a profile that is associated with a reparative phenotype (77). This functional activity is supported by a multitude of studies demonstrating that anti-inflammatory M2-like macrophages are induced in various models of pancreatic injury and inflammation and operate to promote β-cell survival, proliferation, and maintenance of functional β-cell mass, through the release of protective factors, such as IGF-1, epidermal growth factor (EGF), transforming growth factor (TGF), platelet-derived growth factor (PDGF), and Wnt3a (78).

Collectively, these observations demonstrate that macrophages exhibit distinct expression profiles and associated functional activities that are highly specialized in response to the islet microenvironment. As a result, the crosstalk between β-cells and macrophages plays a central role in both the maintenance of islet homeostasis and the initiation/perpetuation of inflammation. Accordingly, the macrophage / β-cell crosstalk can dictate autoimmunity and β-cell destruction, or conversely, immune tolerance and preservation of β-cell mass and function. Manipulating this metabolic conversation offers a unique and new strategy to directly affect β-cell survival and preservation of β-cell mass, and thus prevent the islet inflammatory events underpinning T1D and T2D.

## 5 Using the immune regulatory mechanisms of helminths to positively modulate macrophage and β-cell crosstalk for diabetes prevention

The inverse correlation between the prevalence of infection with helminths and the incidence of immune-mediated metabolic disease has been explained by the ‘old friends hypothesis’. This proposes that the coexistence of helminths and their human hosts over millennia has enabled them to potently regulate the mammalian immune system and skew responses to be anti-inflammatory/tolerogenic/reparative (79–81). This phenomenon affords mutual benefit by promoting longevity and tissue integrity for helminth and host, respectively.

Consequently, the elimination of helminths from human populations, due to enhanced sanitation practices, has also removed their regulatory influence on host immune responses. The result of this is the aberrant activation of inflammatory pathways, thereby increasing the incidence of immune-mediated diseases, such as T1D and T2D. Given these observations, live helminth infection, or the administration of their ES products, are being actively investigated for therapeutic potential (82–84).

### 5.1 Helminths are potent modulators of macrophage phenotype and function

To have sufficient time to mature and reproduce, thereby completing their lifecycle, helminths must establish chronic infections within their mammalian hosts. Accordingly, helminths have developed elegant mechanisms to educate the host’s immune system to tolerate their presence, and therefore support their prolonged survival. Thus, all helminth parasites typically drive the immune response of their hosts towards a predominantly anti-inflammatory phenotype and suppress the development of a pro-inflammatory immune response (85). Reflecting this bias in immune cell activation, helminth infection is commonly associated with an increase in Th2 cytokines, such as IL-4 and IL-13, and a reduction in Th1 cytokines, such as IL-12 and IFNγ (85–87).

Within the immune response to helminth infection, macrophages have emerged as the dominant innate immune cells playing a central role in controlling the development of the adaptive immune response, and the pathological outcomes of infection. During parasite infection, macrophages primarily display an anti-inflammatory M2-like phenotype, as characterized by the expression of specific effector molecules, such as arginase-1 (Arg1), Ym1 and resistin-like molecules (RELM). The primary functional role for these cells is the mediation of tissue repair mechanisms (9). Arg1 metabolizes L-arginine into polyamines, urea and L-orthinine, which contributes to collagen synthesis, fibrosis, and wound healing (88, 89). This consumption of L-arginine can also inhibit NO synthesis, as it is the same substrate required by inducible nitric oxide synthase (iNOS) (90), thereby regulating inflammatory activity. Similarly, Ym1 and RELMα are both associated with wound healing, with the former also directly regulating tissue repair (91–93), and the latter mediating collagen deposition and vascular stability (94, 95). In addition to these characteristic markers, helminth induced M2-like macrophages secrete growth factors, such as IGF1, vascular endothelial growth factor (VEGF) and PDGF, which orchestrate collagen deposition, angiogenesis and the recruitment, activation, and proliferation of reparative cells, such as fibroblasts and endothelial cells (9, 96). Besides this functional activity, the M2-like macrophages also regulate excessive inflammation by dampening pro-inflammatory macrophages (9). Thus, as a combined effect, the modulation of macrophage phenotypes during helminth infections acts to minimize/prevent excessive inflammatory immune responses within the host and to repair tissue damage caused by the migratory and feeding activities of the parasite (85).

The modulation of macrophage activities must be mediated by the molecules that are actively secreted by the parasites, and generally referred to as ES products. The ES products from all parasites are heterogenous mixes of proteins, glycoproteins and lipids, which mimic the modulation of immune responses induced by parasite infection *per se*, thus supporting the notion that these ES molecules hold considerable immune modulatory power (97). Accordingly, the ES products of several parasites have been mined to characterize the individual constituent molecules with a capacity to interact with, and modulate, the phenotype/function of host macrophages (**Table 1**).

**Table 1:** Helminth-derived products and molecules that modulate macrophage phenotype and function.

Helminth	Helminth-derived product	Macrophage cell type	Biological effect on macrophage	Reference
*Acanthocheilonema viteae*	Excretory/secretory products (ES)-62 purified from adult fluke	Ex vivo murine (BALB/c and 129) peritoneal macrophages	Suppressed IFNγ/LPS-induced production of IL-12, IL-6, and TNF via interaction with TLR-4	(71, 72)
	Cysteine protease inhibitor (AvCystatin); *E. coli* recombinant	Murine (C57BL6 and BALB/c) peritoneal macrophages	Induced regulatory/M2-like phenotype and increased production of IL-10, via activation of MAPK signalling pathways	(73, 74)
*Ancylostoma ceylanicum*	*A. ceylanicum* metalloprotease 2 (Ace-MTP-2); *E. coli* and *P. pastoris* recombinant	Human monocyte cell line (THP-1) and THP-1 differentiated macrophages	Enhanced the secretion of TNF and induced the release IFNγ in LPS-exposed macrophages	(75)
*Ascaris lumbricoides*	Lumbricoides protein with cysteine protease inhibitor activity (AI-CPI); *E. coli* recombinant	Murine macrophage cell line (RAW 264.7)	Inhibited macrophage secretion of IL1β, TNF, IFNγ and IL-6 following LPS exposure. Enhanced production of IL-10 and TGFβ, suggesting polarisation to an anti-inflammatory M2 phenotype	(76)
*Ascaris suum*	Adult body fluid (ABF)	Human monocyte-derived macrophages (from peripheral blood mononuclear cells)	Inhibited secretion of LPS-induced TNF and IL-6	(77)
*Brugia malayi*	Macrophage migration inhibitory factor (MIF); *E. coli* recombinant	Murine (C57BL6) bone marrow derived macrophages, murine (BALB/c) peritoneal macrophages	Synergized with IL-4 to induce M2-like macrophages expressing key markers (Arg1, RELMα, Ym1 and mannose receptor)	(78)
		Murine (BALBc) peritoneal macrophages	Increased macrophage expression of Ym1	(79)
	Abundant larval transcript (ALT); *E. coli* recombinant	Murine (C57BL6 and CBA) bone marrow derived macrophages, murine macrophage cell line (J774)	Increased expression of SOCS-1 and GATA-3 which are both associated with polarization to an anti-inflammatory phenotype and/or inhibition of pro-inflammatory macrophages	(80)
*Clonorchis sinensis* (Cs)	Type 1 cystatin (CsStefin-1); *E. coli* recombinant	Murine (C57BL6) spleen and mesenteric lymph node derived macrophages	Induced IL-10 secreting macrophages in the spleen and mesenteric lymph nodes, which were associated with reduction in intestinal inflammation	(81)
	Host defence molecule (CsMF6p/HDM); *E. coli* recombinant	Murine macrophage cell line (RAW 264.7)	Induced pro-inflammatory response associated with M1-like phenotype, such as increased expression of TNF and IL-6	(82)
*Echinococcus granulosus*	Cyst fluid (EgCF)	Murine peritoneal macrophages and murine macrophage cell line (RAW 264.7)	Suppressed LPS-induced TNF, IL-12 and IL-6, and increased IL-10	(83)
*Echinococcus multilocularis*	*E. multilocularis* miR-71 (emu-miR-71)	Murine macrophage cell line (RAW 264.7)	Inhibited nitric oxide release from macrophages	(84)
*Fasciola hepatica*	Peroxiredoxin (Prx/Trx); *E. coli* recombinant	Murine (BALB/c) peritoneal macrophages, murine macrophage (RAW 264.7) cell line	Induced markers (Arg1, Ym1, Fizz1) associated with an M2-like phenotype and promoted secretion of IL-10	(85)
	Native fatty acid binding protein (Fh12) purified from adult fluke extract	Human monocyte derived macrophages	Induced markers (Arg1, Ym1) associated with an M2-like phenotype, promoted secretion of IL-10 and downregulated production of NO, TNF, IL1β and IL-12. Effects are likely mediated via TLR4	(86)
		Murine (C57BL6) bone marrow derived macrophages	Suppressed LPS-induced production of TNF, IL1β, IL-12 and IL-6 and inhibited TLR4 activation	(87)
	Fatty acid binding protein (Fh15); *E. coli* recombinant	Murine (C57BL6) bone marrow derived macrophages	Suppressed LPS-induced production of TNF and IL1β, and inhibited TLR4 activation	(88)
	Native glutathione S-transferase (nFhGST) isolated from adult fluke soluble extract	Murine (C57BL6) bone marrow derived macrophages	Suppressed LPS-induced NF-κB-dependent production of TNF and IL1β	(89)
	Cathepsin-L1 (FheCL1); *P. pastoris* recombinant	Ex vivo murine (BALB/c) peritoneal macrophages	Suppressed TLR3-dependent cytokine production (IL-6, IL-12 and TNF) induced by LPS, via cleavage of TRIF	(90)
	Transforming growth factor-like molecule (FhTLM); *E. coli* recombinant	Bovine blood derived macrophages	Induced regulatory phenotype expressing increased levels of IL-10, Arg1, PD-L1 and mannose receptor, along with decreased levels of IL-12 and NO	(91)
	Helminth defence molecule-1 (FhHDM-1); synthetic molecule	Murine (C57BL6) bone marrow derived macrophages, ex vivo non-obese diabetic mice peritoneal macrophages	Suppressed LPS-induced production of TNF, prevented activation of NLRP3 inflammasome via inhibition of lysosomal vATPase, thereby suppressing production of IL1β	(92–94)
	Novel omega-class glutathione transferase (GSTO2); *E. coli* recombinant	Murine macrophage cell line (RAW 264.7)	Decreased expression of IL-6, IL1β, IFNγ and TNF in LPS-exposed macrophages and increased expression of IL-10 and TGFβ	(95)
*Heligmosomoides polygyrus*	*H. polygyrus* derived extracellular vesicles	Murine (C57BL6 and BALB/c) bone marrow derived macrophages, murine macrophage cell line (RAW 264.7)	Suppressed activation of both pro- and anti-inflammatory macrophages, leading to decreased levels of IL-6, IL-12, TNF and CD206, Ym1 and RELMα, respectively	(96)
*Hymenolepis dimimnuta*	*H. dimimnuta* antigen (HdAg)	Murine (BALB/c) bone marrow derived macrophages	Suppressed LPS-induced release of TNF and IL1β by promoting IL-10 signalling	(9)
*Nippostrongylus brasilliensis*	Acetylcholinesterase (AChE); expressed and delivered via *T. musculi*	Murine (BALB/c) peritoneal macrophages	Promoted M1-like macrophages with increased NO and lowered arginase activity, suggesting inhibition of an M2-like phenotype	(97)
*Schistosoma mansoni*	Soluble egg antigen (SEA)	Human macrophages differentiated from monocytes (isolated from human volunteers)	Induced a mix of pro- and anti-inflammatory characteristics; increased expression of IL-10, TNF, IL-12 and TGFβ	(98)
	Schistosomal-derived lypophosphatidylcholine (LPC)	Murine (C57BL6) bone marrow derived and peritoneal macrophages	Induced M2-like macrophages as evidenced by increased Arg1, TGFβ and IL-10	(99)
	Omega 1 (ω1) derived from SEA	Ex vivo murine (C57BL6) peritoneal macrophages	Induced IL1β secretion in peritoneal macrophages stimulated with toll-like receptor 2 ligand	(100)
	Omega 1 (ω1); recombinant, purified from human embryonic kidney 293 cells	Murine (C57BL6) macrophages derived from epididymal white adipose tissue	Promoted IL-33 secretion, leading to polarisation to an anti-inflammatory phenotype	(101)
	Immunomodulatory molecule (Sm16/SPO-1/SmSLP); *P. pastoris* recombinant and synthetic molecule	Murine (BALB/c) bone marrow derived macrophages and human THP-1 differentiated macrophages	Decreased IL-6 and TNF induced by LPS exposure, but increased the same pro-inflammatory cytokines when administered alone	(102)
*Schistosoma japonicum* (Sj)	CP1412 protein; *E. coli* recombinant	Murine macrophage cell line (RAW 264.7)	Increased expression of CD206, Arg1 and IL-10 associated with polarization to an M2-like phenotype	(103)
	Sj16 protein; *E. coli* recombinant	Murine (BALB/c) peritoneal macrophages	Downregulated LPS-induced TNF expression and upregulated IL-10, associated with polarization to an anti-inflammatory phenotype	(104)
	*S. japonicum* derived extracellular vesicles	Murine macrophage cell line (RAW 264.7)	Promoted polarization to M1-like phenotype, with increased expression of CD16/32, iNOS and TNF	(105)
*Taenia crassiceps*	Excretory/secretory products (TcES)	Murine (BALB/c) bone marrow derived macrophages	Decreased IL-6, IL-12 and TNF, and increased IL-10 in LPS exposed macrophages	(106)
*Taenia pisiformis*	Isolated exosome-like vesicles derived from ES	Murine macrophage cell line (RAW 264.7)	Induced production of IL-4, IL-6, IL-10, IL-13 and Arg1, and decreased expression of IL-12, IFNγ and iNOS	(107)
*Toxocara canis*	Excretory/secretory products (ES)	Murine (C57BL6) peritoneal macrophages	Promoted TNF secretion, decreased IL1β and inhibited IL-6 initially followed by continuous increase over time. Induced expression of inflammatory NFκB	(108)
*Trichinella spiralis*	*T. spiralis* excretory/secretory antigens	Murine macrophage cell line (RAW 264.7)	Decreased IL-12 and TNF following LPS exposure, and promoted IL-10 secretion	(109)
		Murine macrophage cell line (J774A.1)	Inhibited TNF, IL1β, IL-6 and IL-12 production following LPS exposure, and promoted IL-10, TGFβ and Arg1	(110)
		Murine (C57BL6) peritoneal macrophages and murine macrophage cell line (RAW 264.7)	Attenuated colitis by promoting M2-like macrophage polarization, as evidenced by increased CD206 and Arg1	(111)
	*T. spiralis*-specific 53 kDA glycoprotein (rTsP53); *E. coli* recombinant	Murine (BALB/c) colon macrophages	Inhibited colitis by promoting polarization to an anti-inflammatory phenotype, as evidenced by increased Arg1 and Fizz1 markers. Increased IL-10 and TGFβ, and decreased IL-6 and TNF	(112)
		Murine (BALB/c) bone marrow derived and peritoneal macrophages	Attenuated sepsis through promotion of M2-like macrophage polarization, associated with increased Arg1 and Fizz1, and reduced iNOS	(113)
	*T. spiralis* novel statin (rTsCstN); *E. coli* recombinant	Murine (BALB/c) bone marrow derived macrophages	Decreased pro-inflammatory IL1β, IFNγ, TNF and iNOS following LPS exposure and inhibits macrophage antigen presentation	(114)
	*T. spiralis* cystatin (Ts-Cys); *E. coli* recombinant	Murine (BALB/c) bone marrow derived macrophages	Promoted macrophage polarization from pro- to anti-inflammatory phenotype by inhibiting TLR2/MyD88 signal pathway, TNF, IL-6, IL-1β, and increasing mannose receptor expression and TGFβ	(115)
	*T. spiralis* cathepsin B-like protein (rTsCPB); *E. coli* recombinant	Murine (BALB/c) intestinal tissue macrophages	Ameliorated ischemia/reperfusion injury by induction of M2-like macrophages, as evidenced by decrease in M1-like markers and increase in M2-like markers	(116)

Arg1, arginase 1; Ym1, chitinase-like protein 3; Fizz1, Found in inflammatory zone; IL; interleukin, NO; nitric oxide, TNFα; tumour necrosis factor alpha, IL1β, interleukin 1 beta; TLR, toll like receptor; LPS, lipopolysaccharide; NF-κB, nuclear factor kappaligand; iNOS-domain-containing adapter-inducing interferon beta; PD-L1, programmed death-ligand 1; NLRP3, NLR family pyrin domain containing 3; RELMα, resistin-like molecule alpha; SOCS-1, suppressor of cytokine signalling-1; MAPK, mitogen-activated protein kinases; CCL, chemokine ligand; iNOS, inducible nitric oxide synthase; MyD88, myeloid differentiation pathway response.

### 5.2 Regulation of macrophage phenotypes by helminths prevents the development of T1D and T2D

The possibility that macrophages mediate the beneficial effect of helminths in diabetes emerged from experimental studies using mouse models in which the depletion of T-cells, or the genetic ablation of T-cell signaling molecules, failed to impact the protection from disease elicited by infection with *Litomosoides sigmodontis, Heligmosomoides polygyrus*, or *Schistosoma mansoni* (98, 99). Further analysis of immune cell populations within the infected non-diabetic animals showed an increase in the expression levels of M2 macrophage markers in the pancreatic lymph nodes. This finding indicated a central role for M2 macrophages in disease protection (100). Subsequent studies using the ES products of *Fasciola hepatica* and *Taenia crassiceps* confirmed the association between helminth-mediated protection and the recruitment of M2-like macrophages (101, 102). A functional role for macrophages was confirmed by depletion studies, in which the removal of macrophage populations, by the administration of clodronate-liposomes reversed the protective effect of the *T. crassiceps* ES products (102). Albeit, such macrophage ablation studies must be interpreted with caution, as in a recent study, the anti-inflammatory effects of clodronate liposomes in arthritis models were attributed to the modulation of neutrophil effector functions after phagocytosis of liposomes (103).

Analysis of the individual constituents of the *F. hepatica* ES products identified a single protein (FhHDM-1), which mimicked the protective effect of the ES products in preventing both insulitis and hyperglycemia in the non-obese diabetic (NOD) mouse model of T1D (104). While the reduction in disease progression was correlated to the modulation of macrophage activity, in contrast to the administration of the ES products, FhHDM-1-mediated protection was associated with the reduced ability of macrophages to respond to pro-inflammatory ligands, suggesting the regulation of an M1-like phenotype, and indicating multiple mechanisms mediating an overall switch from a predominance of pro-inflammatory to anti-inflammatory macrophages (104).

Helminth-mediated macrophage modulation is also advantageous in the prevention of T2D. Indeed, infection with various helminths, such as *S. mansoni* (105), *Strongyloides venezuelensis*, *L. sigmodontis* (106), *Nippostrongylus brasiliensis* (107), *Trichinella spiralis* (108), and *H. polygyrus* (109, 110), have been shown to exert beneficial effects (such as improvement of glucose tolerance and insulin sensitivity) in various T2D mouse models (**Table 2**). The mechanism of action has been putatively attributed to the activation/recruitment of M2-like macrophage populations within the adipose tissue. These M2-like populations oppose the obesity-induced elevation of pro-inflammatory macrophages and promote an anti-inflammatory environment akin to that observed in lean adipose tissue. Of these helminths, a wide array of molecules derived from *S. mansoni* (105, 111–113) and *L. sigmodontis* (106) similarly provided protection from T2D through the same mechanism (**Table 2**). This helminth-mediated reduction of inflammation in adipocyte tissue and subsequent improvements in glucose homeostasis could also have positive effects on the β-cells, by reducing FFAs levels and hyperglycemia that contribute to β-cell stress and exhaustion (8).

**Table 2:** Helminth infection and helminth-derived products exert protective effects against obesity and type 2 diabetes.

Helminth/product	Infection/dose	T2D model	Macrophage activation	Therapeutic effect	References
*Schistosoma mansoni* infection	Percutaneous infection with 36 larvae, chronic/12wk infection	HFD-induced obese C57BL/6 mice	Promoted M2-like macrophages in white adipose tissue	Reduced weight and fat mass gain, improved glucose tolerance and insulin sensitivity	(117)
*S. mansoni* SEA	i.p. injection with 50μg SEA once every 3d for 4wks				
*S. mansoni*-derived ω1	Recombinant, i.p. injection with 25μg ω1 on 0, 2 and 4d	HFD-induced obese C57BL/6 mice^a^	Promoted M2-like macrophages in adipose tissue dependent on IL-33 release	Reduced weight gain and promoted glucose homeostasis in an IL-33 dependent manner	(101)
*S. mansoni*-derived ω1	Recombinant, 50μg i.p. injection every 3d for 4wks	HFD-induced obese C57BL/6 mice	Promoted M2-like macrophages in white adipose tissue	Reduced weight gain and improved glucose tolerance and insulin sensitivity	(118)
LNFPIII	Synthetic glycan, 25μg i.p. injection twice per wk., 4-6wks after HFD-induced obesity onset	HFD-induced obese C57BL/6 mice	Promoted M2-like macrophages in metabolic tissue	Improved glucose tolerance and insulin sensitivity, partly mediated through M2 macrophage-derived IL-10	(119)
*Litomosoides sigmodontis* infection	Larvae transmitted with blood meal of infected *Ornithonyssus bacoti* mites at 8-10wks of age (1-2wks after HFD onset)	HFD-induced obese BALB/c mice	Promoted M2-like macrophages in epididymal adipose tissue	Improved glucose tolerance, LsAg reduced inflammatory immune response and enhanced insulin signalling	(120)
*L. sigmodontis* antigen (LsAg)	LsAg derived from adult worms, 2μg i.p. injection daily at 8-10wks or 12-14wks of HFD	HFD-induced obese C57BL/6 mice			
*Nippostrongylus brasiliensis* infection	Subcutaneous infection with 500 larvae once every 4wks for total of 3 infections for HFD mice, at 10 and 17wks of age for RIP2 Opa1KO mice	HFD-induced obese RIP2-Opa1KO mice or C57BL/6 mice	Promoted M2-like macrophages in epididymal adipose tissue	Reduced weight gain and improved glucose tolerance	(121)
*Trichinella spiralis* infection	Oral infection with 400 larvae, after 4wks on HFD	Ob/ob mice and HFD-induced obese C57BL/6 mice	Promoted M2-like macrophages in adipose tissue	Improved glucose tolerance and insulin sensitivity	(122)
*Heligmosomoides polygyrus* infection	Oral infection with 200 larvae after ~4wks on HFD	HFD-induced obese C57BL/6 mice	Promoted M2-like macrophages in gonadal fat tissue	Reduced weight gain, improved glucose tolerance and increased browning of white adipose tissue. Adoptive transfer of *H. polygyrus* induced M2 macrophages similarly attenuated HFD-induced obesity	(123)
*Heligmosomoides polygyrus* infection	Oral infection with 200 larvae at 10-12wks of age	HFD-induced obese C57BL/6 mice	Promoted M2 -like macrophages in adipose tissue	Reduced weight gain and improved glucose tolerance	(124)

wk, week; SEA, soluble egg antigen; IP, intraperitoneal; HFD, high fat diet; ω1, omega-1; LNFPIII, Lacto-N-fucopentaose III; LsAg, Litomosoides sigmodontis antigen; Lepr^db/db^, diabetes (db) mutation of the leptin receptor, ^a^Various mouse models were crossed to C57BL/6 background for mechanistic studies, including T1/ST2-deficient mice, IL-33 citrine reporter mice, and IL-33 deficient mice. CD206^-/-^ and Rora^sg/sg^ were also used.

### 5.3 Helminth-mediated changes to macrophages may alter the crosstalk with β-cells

While the aforementioned studies clearly demonstrate a critical role for macrophages in the protective mechanisms of helminths against diabetes development, most reports simply concluded that the beneficial effects were attributable to a switch in the predominant inflammatory phenotype of macrophage. However, we propose that the changes to macrophages elicited by helminths, and their ES products, are also exerting a protective effect through communication with β-cells. Indeed, the M2-like macrophages induced by helminth infection exhibited increased expression levels of IGF1 (114). Additionally, ablation of IGF1 signaling in macrophages has been shown to disrupt endocrine IGF1-mediated signaling, resulting in a significant increase in insulin resistance in mice (114). Although a specific link to β-cell function was not explored in these studies, it has been reported that macrophages are the primary source of IGF1 in pancreatic islets, and that these cells function to enhance insulin secretion from β-cells (77). Furthermore, other growth factors (such as TGFβ1 and EGF) secreted by M2-like macrophages have been shown to enhance the survival and proliferation of β-cells through the increased expression levels of SMAD7 (115).

Supporting the beneficial effect of M2-anti-inflammatory macrophages in diabetes prevention, is the helminth-mediated regulation of pro-inflammatory macrophages. Under diabetic conditions, phagocytosis of apoptotic β-cells by macrophages generates ROS, leading to inflammasome activation, and the secretion of pro-inflammatory cytokines (notably IL-1 β), all hallmarks of an M1-like phenotype (60). As mentioned above, these macrophages have deleterious effects on β-cells by impairing glucose-stimulated insulin secretion, inducing apoptosis, and causing β-cell dedifferentiation. Further crosstalk between pro-inflammatory macrophages and pathogenic β-cells work in concert to drive inflammation and disease. Indeed, macrophages within the islets of NOD mice have been found to exhibit M1-like features (10), and depletion of these macrophages prevented T1D onset (116). Furthermore, typically, M1-like macrophages exhibit decreased phagocytic ability, as observed in diabetes-prone NOD mice (125) and humans (126), which could initiate and exacerbate inflammation because apoptotic β-cells are not efficiently cleared. Helminths can hinder this deleterious activity of pro-inflammatory macrophages (through inhibition of the inflammasome and/or secretion of pro-inflammatory cytokines (127)) or skew macrophages towards an anti-inflammatory M2-like phenotype. Consequently, the destructive pathway of macrophage/β-cell communication would be inhibited, the perpetuation of β-cell destruction prevented, and anti-inflammatory pathways conducive with preservation of β-cell function and mass, and disease prevention, would prevail.

## 6 Helminths modulate whole body metabolism independently of immune regulation

In recent years, it has become evident that infection with helminth parasites triggers a remodelling of the systemic metabolism of the host, which is demonstrated by altered production of pancreatic hormones, namely incretins and adipokines (128). Additionally, there is a shift in metabolic pathways in both infected tissue and in organs beyond the location of the parasite (17). The current theoretical framework suggests that this metabolic shift is required to direct the host immune status, and eventually impacts cells and organs that are not directly involved in the immune response (17, 129). This rewiring of the host’s metabolism commonly manifests as a significant increase in insulin sensitivity in non-diabetic helminth infected humans and mice (114, 117).

### 6.1 Helminth derived molecules interact with endocrine cells

We have recently shown that the *F. hepatica* FhHDM-1, which was previously shown to prevent T1D via modulation of macrophage activity, can also interact directly with pancreatic β-cells to modulate their activities (120, 121). After interaction through a currently unknown binding partner, FhHDM-1 activates the PI3K/Akt signaling pathway in β-cells. This consequently promotes β-cell survival and function, without enhancing proliferation, and inhibits pro-inflammatory cytokine induced apoptosis in both a NOD-derived (diabetogenic) β-cell line and in human islets (121).

Due to the focus on the contributory role for immune modulation in the protective effect mediated by helminths, there are limited additional studies specifically investigating the interactions of helminths, and their ES products, with β-cells. However, there is evidence supporting this premise. As mentioned previously, pancreatic β-cells express TLRs, which can modulate β-cell viability, influence insulin homeostasis (122), and contribute to T1D initiation upon interaction with high-mobility group box 1 (HMGB1) (123). Since the molecules secreted by helminths can interact with TLRs, these receptors may allow direct helminth regulation of β-cells (124). Furthermore, given the diversity of helminth molecules (including unique glycoproteins) it is plausible that helminth-derived molecules can directly modulate metabolic processes through glycan activation of metabolic cell receptors and C-type lectins (119). For example, the glycan, lacto-N-fucopentaose III (LNFPIII), which is the predominant polylactosamine sugar found on secreted egg antigens of *S. mansoni* exerted a similar dual effect as FhHDM-1. Under pre-T2D conditions (obesity), LNFPIII improved glucose tolerance and insulin sensitivity, by modulating pro-inflammatory responses and by directly inhibiting lipogenesis in hepatocytes, thereby ameliorating hepatic steatosis (112). Similarly, the T2 RNase ω1 secreted from the same helminth, promoted metabolic homeostasis in a murine model of diet induced T2D. In this case, ω1 was found to enhance adipocyte expression and secretion of IL-33, likely through interaction with the mannose receptor (111). Several other studies have reported similar direct helminth modulation of non-immune, metabolically active cells (8, 107, 118) (**Table 3**), predominantly adipocytes and hepatocytes, which communicate with, and influence the function of, β-cells. Indeed, adipocytes secrete several adipokines, such as leptin, adipsin and adiponectin, that contribute to regulation of β-cell insulin secretion and proliferation (132). Furthermore, extracellular vesicles (EVs) secreted by healthy adipocytes have been shown to promote normal β-cell physiology and provided protection from palmitate or pro-inflammatory cytokine induced death. Conversely, EVs derived from obese adipocytes led to β-cell dysfunction and death. Similar crosstalk has been observed between β-cells and hepatocytes (133). Hepatocytes secrete EVs and proliferative factors, such as IGF1, that contribute to compensatory β-cell hyperplasia in conditions of obesity/T2D. Moreover, under this inflammatory environment, pancreatic β-cell dysfunction leads to impaired insulin secretion that normally modulates glucagon release, thus leading to elevated glucagon levels. In turn, this exacerbates the increased glucose output from the liver associated with insulin resistance and induces production of kisspeptin1 in hepatocytes that can further impair β-cell insulin secretion (132). Therefore, helminth-mediated changes to adipocytes and hepatocytes would be expected to have significant knock-on effects to the β-cells.

**Table 3:** Helminths and helminth derived products directly affect endocrine cells.

Helminth/product	Infection/dose	Interaction with non-immune cells	Reference
*Fasciola hepatica*; FhHDM-1	Synthetic molecule, total of six 10μg i.p. injections given on alternate days from 4wks of age in NOD mice, 10μM dose for isolated β-cells	Promoted β-cell survival and function without enhancing proliferation, and inhibited pro-inflammatory cytokine-induced apoptosis, via activation of PI3K/Akt signalling pathway	(130)
*Schistosoma mansoni;* LNFPIII	Synthetic molecule, 25μg i.p. injection twice per wk.	Suppressed lipogenesis in hepatocytes and protected against hepatic steatosis by upregulating bile acid sensing nuclear receptor *Fxr-α* signalling	(119)
*Schistosoma mansoni;* ω1	Recombinant, i.p. injection of 25μg on 0, 2 and 4d	Enhanced adipocyte expression and secretion of IL-33, likely through interaction with the mannose receptor	(101)
*Nippostrongylus brasiliensis* infection	Subcutaneous infection with 500 larvae once every 4wks for total of 3 infections for HFD mice	Suppressed lipogenesis in hepatocytes and ameliorated hepatic steatosis, and decreased expression of key glucose transporters in intestinal cells, which could normalise glucose homeostasis under conditions of obesity	(121)
*Schistosoma japonicum* SEA	20μg/ml dose used on hepatic stellate cells	Impaired NF-κB activation in hepatic stellate cells, thereby inhibiting TNF-induced pro-fibrotic IL-34 and progression of hepatic fibrosis	(131)
*Litomosoides sigmodontis* infection	Unpublished data	Downregulated adipogenesis related genes such as PPARγ and C/EBPα in epididymal adipose tissue	(5)
*Litomosoides sigmodontis* antigen	Unpublished data	Reduced differentiation of pre-adipocyte cell line (3T3-L1) into mature adipocytes, suggesting suppression of adipogenesis	(5)

FhHDM-1, Fasciola hepatica helminth defence molecule-1; wk, week; NOD, non-obese diabetic; LNFPIII, lacto-N-fucopentaose III; ω1, omega-1; HFD, high fat diet; SEA, soluble egg antigen; IL, interleukin; NF-κB, nuclear factor kappa B; PPARγ, peroxisome proliferator-activated receptor gamma; C/EBPα, CCAAT/enhancer binding protein alpha.

### 6.2 Helminths alter the gut-islet axis

Extensive analyses of the human microbiome have revealed that the gut microbiota is deeply interconnected with diabetes, with alterations to the populations of species in the gut impacting several metabolic effects and immune response processes (134, 135). It is widely acknowledged that gastrointestinal factors, such as glucagon like peptide (GLP)-1 and gastric inhibitory polypeptide (GIP), interact directly with β-cells to induce proliferation, and enhance resistance to apoptosis, thereby increasing/maintaining β-cell mass (136, 137). In addition, short chain fatty acids (SCFA) released by gut microbiota modulate the function and survival of β-cells (132). Thus, any changes to the bacteria populating the gut will subsequently alter the metabolic activity of β-cells within the pancreas.

As the gut mucosa hosts the largest population of macrophages in the human body (138), it is not surprising that SCFA released by gut microbiota also influences their functional phenotypes. For example, it has been reported that an increased abundance in *Bacteroides fragilis*, *Lactobacillus* spp. *and Clostridia* class induce the polarization of anti-inflammatory M2-like macrophages (139, 140). In contrast, the presence of *Enterococcus faecalis* polarizes colonic macrophages towards a pro-inflammatory phenotype (141). However, these effects are not confined to the intestinal microenvironment, as it has been shown that butyrate produced by gut microbiota induces the expression of mouse β-defensin 14 in pancreatic islets, which in turn drives the activation of regulatory macrophages and prevents the development of T1D in a mouse model (130).

Several studies of human and murine helminth infection have demonstrated that the presence of the parasites alters both the abundance and diversity of gut microbiomes (142, 143). More recently it has been demonstrated that these changes mediate a beneficial effect in models of T2D diabetes. Infection of mice with either *S. venezuelensis* (144) or *Nippostrongylus brasiliensis* (145) resulted in significant compositional changes in gut microbiota, most notably by increasing *Lactobacillus* spp., a bacterial species which has been assessed as a beneficial probiotic for diabetes (131). In both cases, these changes in gut microbiota resulted in improved insulin signaling and sensitivity. Although a specific effect on β-cells was not assessed in these studies, increased expression of M2 macrophage markers were found in adipose tissue, liver, and gut.

## 7 Concluding remarks

**Figure 4:**
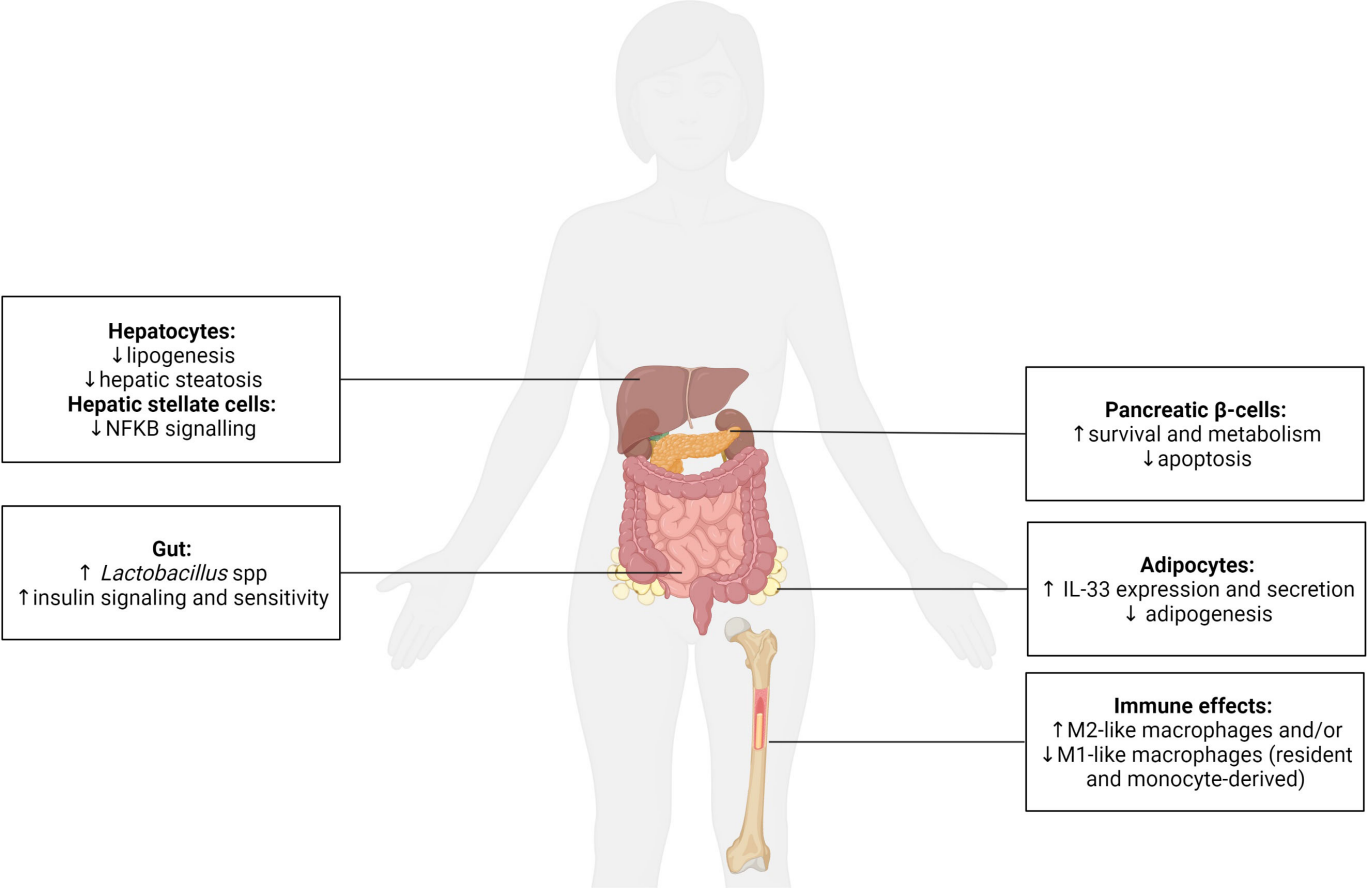
The systemic effect of helminths and their secreted molecules on the function of metabolic and immune cells. Helminths are recognized as potent immune modulators, typically promoting the polarization of macrophages (resident and monocyte-derived) to an anti-inflammatory/regulatory M2-like phenotype and/or inhibiting a pro-inflammatory M1-like phenotype. This immune regulation has been shown to be beneficial for type 1 and type 2 diabetes. Helminths also positively impact endocrine cells and have been demonstrated to (i) enhance pancreatic β-cell survival and metabolism, (ii) promote adipocyte expression of anti-inflammatory IL-33 and inhibit adipogenesis, (iii) reduce lipogenesis and prevent hepatic steatosis within hepatocytes, and decrease pro-inflammatory nuclear factor kappa (κ) B signaling in hepatic stellate cells, and (iv) increase the presence of probiotic *Lactobacillus* spp. in the gut, which promotes insulin signaling/sensitivity. Created with BioRender.com.

T1D and T2D are characterised by a progressive dysfunction and loss of the insulin-producing β-cells in the pancreatic islets leading to insulin deficiency. Despite this knowledge, none of the currently prescribed anti-diabetic agents effectively target the maintenance of functional β-cell mass. In T1D, the focus remains on blocking autoreactive immune cells and their mediators. However, diabetes reversal is not achieved, protection of residual β-cell mass is short-term, and global immune suppression is induced (146–148). Due to the scarcity of organ donors, significant β-cell loss during islet preparation, and allogenic and recurrent autoimmune reactions, which mediate β-cell death post-transplant, islet transplantation is not a realistic cure for T1D. In T2D, therapeutic options, such as dipeptidyl peptidase-4 (DPP-4) inhibitors, incretin-based drugs, and GLP-1 analogues, target β-cell function, but not all patients are responsive and approximately 50% ultimately require daily insulin injections due to β-cell exhaustion and death (149). Clearly, alternate, more effective strategies, are urgently required. By regulating the activities of islet macrophages and β-cells (and other endocrine cells), helminth parasites shape their crosstalk (**Figure 4**). This offers a unique opportunity to exploit helminths’ mechanisms for survival in their mammalian hosts to establish an environment that preserves β-cell mass and function and thus offers the potential as a cure for both T1D and T2D.

## Author contributions

IC, BO’B and SD conceived and planned the review. IC conducted the literature search, designed and wrote the first draft of the manuscript. BO’B and SD contributed to the writing and editing of the final manuscript. All authors contributed to the article and approved the submitted version.
